# (*E*)-1-(Anthracen-9-yl)-3-(2-chloro-6-fluoro­phen­yl)prop-2-en-1-one: crystal structure and Hirshfeld surface analysis

**DOI:** 10.1107/S2056989016005028

**Published:** 2016-04-08

**Authors:** Amzar Ahlami Abdullah, Nur Hafiq Hanif Hassan, Suhana Arshad, Nuridayanti Che Khalib, Ibrahim Abdul Razak

**Affiliations:** a School of Physics, Universiti Sains Malaysia, 11800 USM, Penang, Malaysia

**Keywords:** crystal structure, chalcone, hydrogen bonding, Hirshfeld surface analysis

## Abstract

In the title compound, C_23_H_14_ClFO, the enone moiety adopts an *E* conformation. The dihedral angle between the benzene and anthracene ring is 63.42 (8)° and an intra­molecular C—H⋯F hydrogen bond generates an *S*(6) ring motif. In the crystal, mol­ecules are arranged into centrosymmetric dimers *via* pairs of C—H⋯F hydrogen bonds. The crystal structure also features C—H⋯π and π–π inter­actions. Hirshfeld surface analysis was used to confirm the existence of inter­molecular inter­actions.

## Abstract 2

In the title compund, the enone moiety adopts an *E* conformation. An intra­molecular C—H⋯F hydrogen bond generates an *S*(6) ring motif. In the crystal, mol­ecules are arranged into centrosymmetric dimers *via* pairs of C—H⋯F hydrogen bonds. The crystal structure also features C—H⋯π and π–π inter­actions. Hirshfeld surface analysis was used to confirm the existence of inter­molecular inter­actions.

## Chemical context   

The biological properties of chalcone derivatives such as anti­cancer (Bhat *et al.*, 2005 ▸), anti­malarial (Xue *et al.*, 2004 ▸), anti-oxidant and anti­microbial (Yayli *et al.*, 2006 ▸), anti­platelet (Zhao *et al.*, 2005 ▸) as well as anti-inflammatory (Madan *et al.*, 2000 ▸) have been studied extensively and developed. As part of our own studies in this area, we hereby report the synthesis and crystal structure of the title compound.
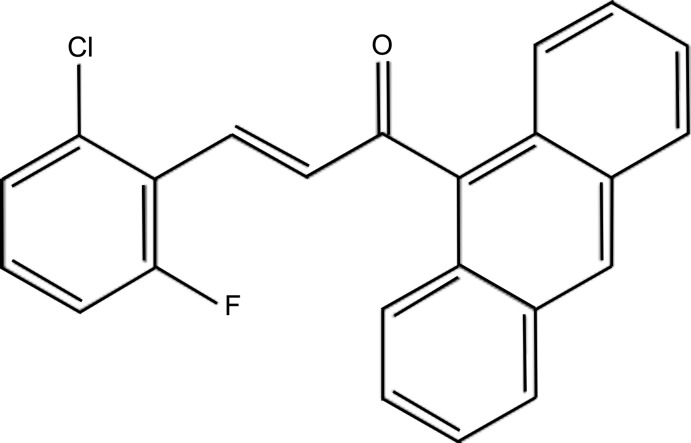


## Structural commentary   

**Figure 1:**
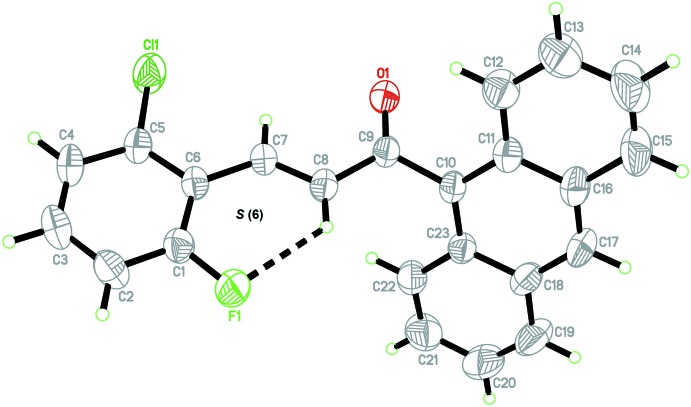
The mol­ecular structure of the title compound, showing 50% probability displacement ellipsoids. The intra­molecular C—H⋯F hydrogen bond is shown as a dashed line.

The mol­ecular structure of the title chalcone is shown in Fig. 1 ▸. The enone moiety (O1/C7–C9) adopts an *E* conformation with respect to the C7=C8 bond. The anthracene ring system (C10–C23) is twisted at the C9–C10 bond from the (*E*)-3-(2-chloro-6-fluoro­phen­yl)acryl­aldehyde moiety [maximum deviation = 0.193 (16) Å for atom O1] with a C8—C9—C10—C23 torsion angle of −61.4 (2)°. The terminal benzene and anthracene ring systems (C1–C6 and C10–C23, respectively) form a dihedral angle of 63.42 (8)°. The least-squares plane through the enone moiety [O1/C7–C9) with a maximum deviation of 0.033 (2) Å for atom C9] makes dihedral angles of 5.62 (13) and 59.18 (12)° with the benzene (C1–C6) and anthracene (C10–C23) rings, respectively. An intra­molecular C8—H8*A*⋯F1 hydrogen bond is observed, generating an *S*(6) ring motif. The bond lengths and angles are comparable with those in previously reported structures (Razak *et al.*, 2009 ▸; Ngaini *et al.*, 2011 ▸).

## Supra­molecular features   

**Figure 2:**
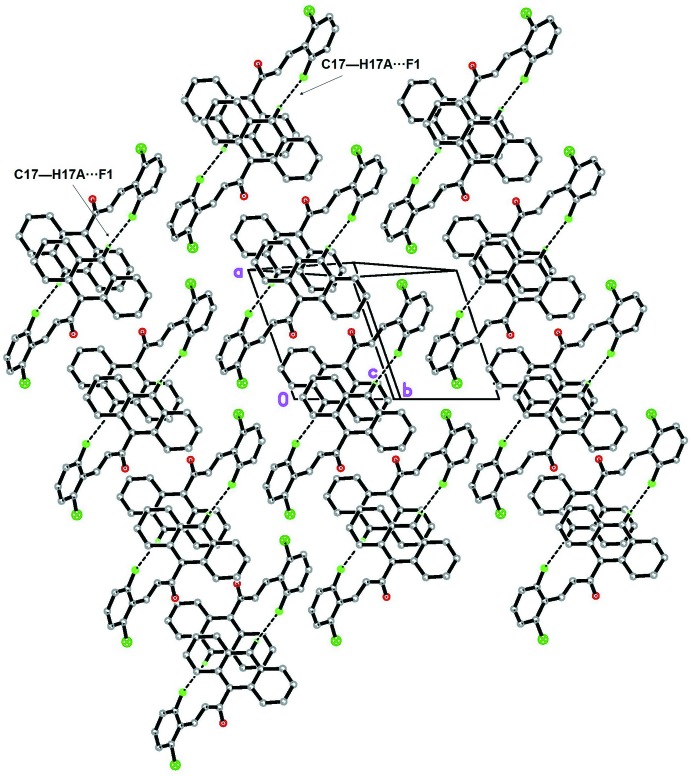
The crystal packing showing the mol­ecules arranged into centrosymmetric dimers. The H atoms not involved in the inter­molecular inter­actions (dashed lines) have been omitted for clarity.

**Figure 3:**
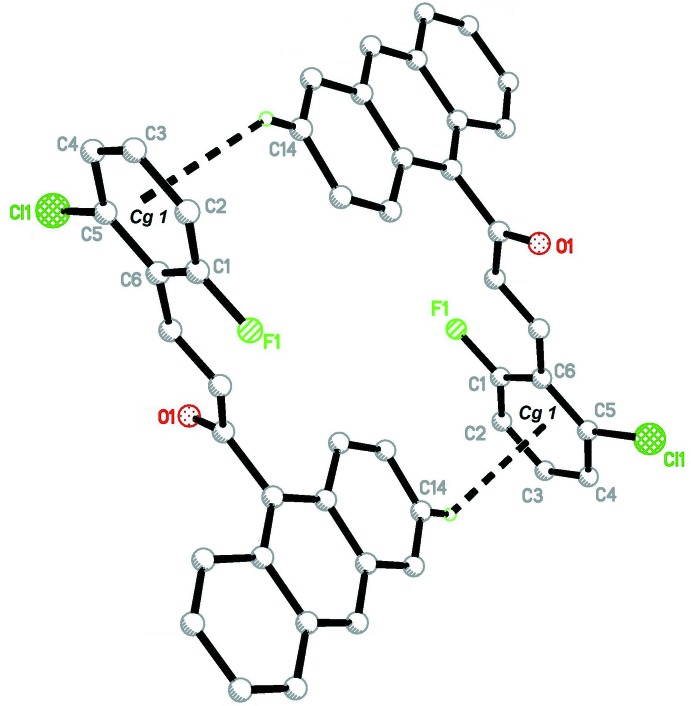
Detail of the crystal structure showing the C14—H14*A*⋯*Cg*1 inter­action where *Cg*1 is the centroid of C1–C6 ring.

In the crystal (Fig. 2 ▸), the mol­ecules are arranged into centrosymmetric dimers *via* pairs of C17—H17*A*⋯F1 (Table 1 ▸) hydrogen bonds. The crystal structure also features C14—H14*A*⋯*Cg*1 (Fig. 3 ▸) and *Cg*1⋯*Cg*1(1 − *x*, −*y*, 1 − *z*) inter­actions [centroid-to-centroid distance = 3.7557 (13) Å; *Cg*1 is the centroid of the C1–C6 ring].

## Hirshfeld surfaces analysis   

**Figure 4:**
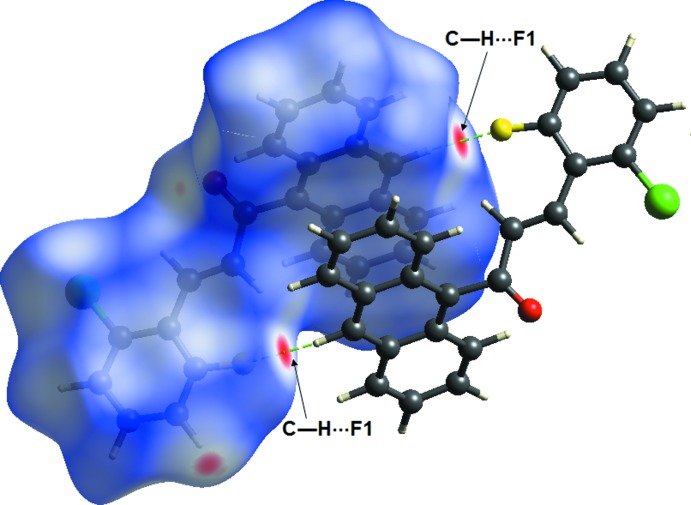
*d*_norm_ mapped on the Hirshfeld surface for visualizing the inter­molecular inter­actions of the title chalcone compound. Dotted lines (green) represent hydrogen bonds.

**Figure 5:**
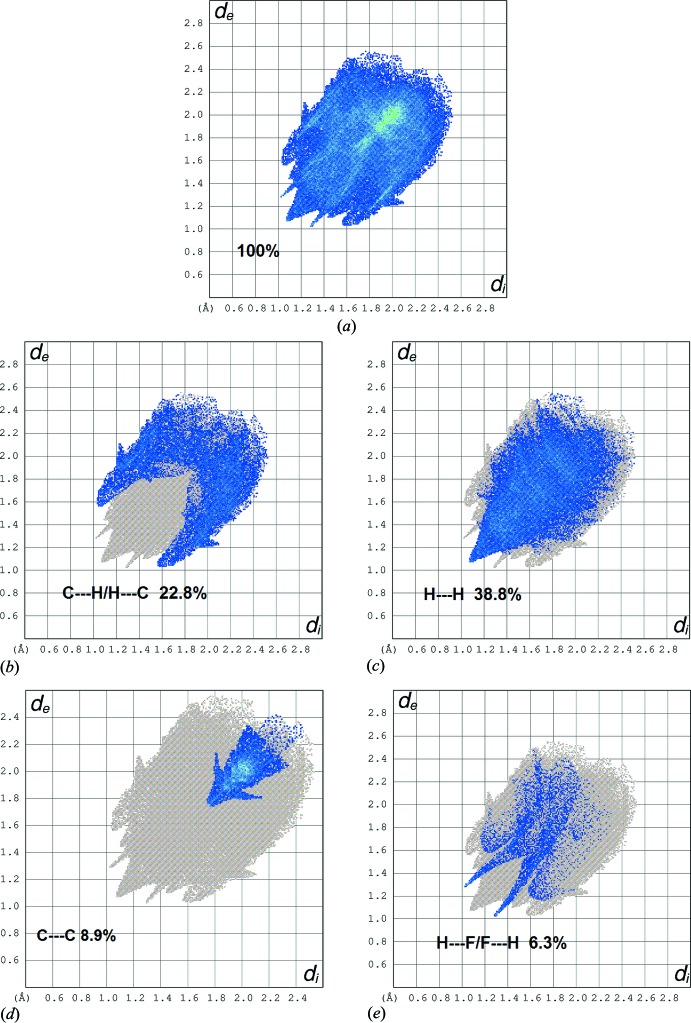
The 2-Dimensional fingerprint plot for the title chalcone compound showing contributions from different contacts.

The inter­molecular inter­actions of the title compound can be visualized using Hirshfeld surface analysis (Wolff *et al.*, 2012 ▸). The Hirshfeld surfaces mapped over *d*_norm_ are shown in Fig. 4 ▸. The 2-D fingerprint plots showing the occurrence of different kinds of inter­molecular contacts are shown in Fig. 5 ▸.

The C17—H17*A*⋯F1 inter­actions are shown on the Hirshfeld surfaces marked with a bright-red spot for short contacts·The H⋯F/F⋯H contacts comprise 6.3% of the total Hirshfeld surface, represented by two symmetrical narrow pointed spikes with *d_e_* + *d_i_* ∼2.3 Å, suggesting the presence of a non-classical C—H⋯F hydrogen bond. The H⋯H contacts are shown on the fingerprint plot as one distinct spike with the minimum value of *d_e_* + *d_i_*. These contacts represent the largest contribution within the Hirshfeld surfaces (38.8%).

**Figure 6:**
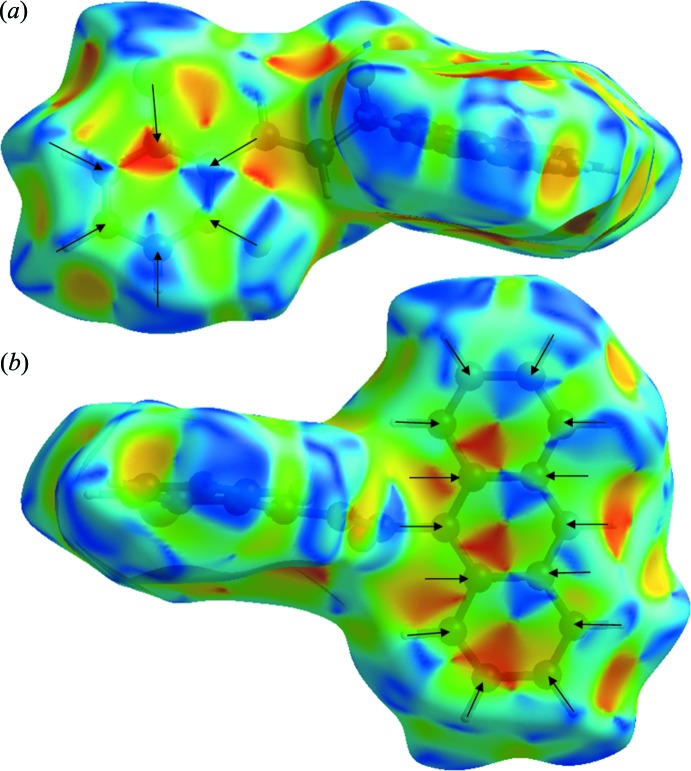
Hirshfeld surface mapped over the shape index of the chalcone compound in (*a*) front view and (*b*) back view.

**Figure 7:**
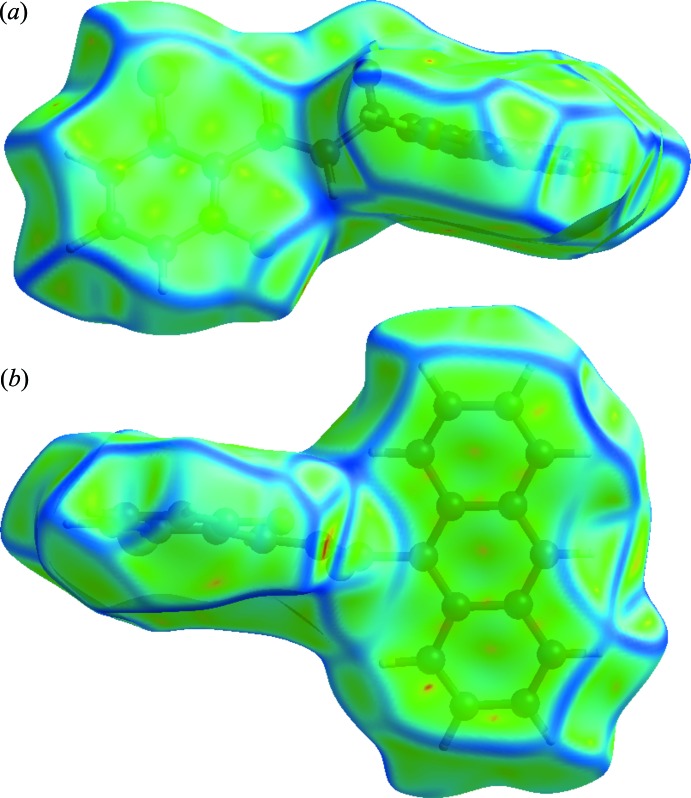
Hirshfeld surface mapped over curvedness of the chalcone compound in (*a*) front view and (*b*) back view.

Significant C—H⋯π inter­actions (22.8%) can be also be seen, indicated by the wings of *d_e_* + *d_i_* ∼2.6 Å on the fingerprint plot. The presence of π–π inter­actions is shown as C⋯C contacts, which contribute 8.9% of the Hirshfeld surfaces. The presence of these inter­actions can also be shown by the Hirshfeld surfaces mapped by shape index (Fig. 6 ▸) and the Hirshfeld surfaces mapped with curvedness (Fig. 7 ▸).

## Synthesis and crystallization   

A mixture of 9-acetyl­anthracene (0.1 mol, 0.11 g) and 2-chloro-6-fluoro­benzaldehyde (0.1 mol, 0.08 g) was dissolved in methanol (20 ml). A catalytic amount of NaOH (5 ml, 20%) was added to the solution dropwise with vigorous stirring. The reaction mixture was stirred for about 5–6 h at room temperature. After stirring, the contents of the flask were poured into ice-cold water (50 ml) and the resulting crude solid was collected by filtration. The compound was dried and purified by repeated recrystallization from acetone solution, forming yellow plates.

## Refinement details   

Crystal data collection and structure refinement details are summarized in Table 2 ▸. All H atoms were positioned geometrically (C—H = 0.93 Å) and refined using a riding model with *U*_iso_(H) = 1.2*U*_eq_(C). The most disagreeable reflections (−1 − 2 4 and −1 1 0) were omitted from the final refinement.

## Supplementary Material

Crystal structure: contains datablock(s) I, global. DOI: 10.1107/S2056989016005028/hb7569sup1.cif

Structure factors: contains datablock(s) I. DOI: 10.1107/S2056989016005028/hb7569Isup2.hkl

Supporting information file. DOI: 10.1107/S2056989016005028/hb7569Isup3.cml

CCDC reference: 1470351

Additional supporting information:  crystallographic information; 3D view; checkCIF report

## Figures and Tables

**Table 1:** Hydrogen-bond geometry (Å, °) *Cg*1 is the centroid of the C1–C6 ring.

*D*—H⋯*A*	*D*—H	H⋯*A*	*D*⋯*A*	*D*—H⋯*A*
C8—H8*A*⋯F1	0.93	2.19	2.808 (2)	123
C17—H17*A*⋯F1^i^	0.93	2.46	3.353 (2)	161
C14—H14*A*⋯*Cg*1^ii^	0.93	2.99	3.712 (3)	136

Symmetry codes: (i) 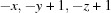; (ii) 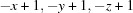.

**Table 2:** Experimental details

Crystal data	
Chemical formula	C_23_H_14_ClFO
*M*_r_	360.79
Crystal system, space group	Triclinic, *P* 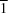
Temperature (K)	296
*a*, *b*, *c* (Å)	9.2846 (9), 9.8777 (10), 10.3624 (11)
α, β, γ (°)	94.364 (2), 113.3517 (19), 92.866 (2)
*V* (Å^3^)	866.63 (15)
*Z*	2
Radiation type	Mo *K*α
μ (mm^−1^)	0.24
Crystal size (mm)	0.43 × 0.39 × 0.11

Data collection	
Diffractometer	Bruker SMART APEXII DUO CCD
Absorption correction	Multi-scan (*SADABS*; Bruker, 2009 ▸)
*T*_min_, *T*_max_	0.905, 0.974
No. of measured, independent and observed [*I* > 2σ(*I*)] reflections	15408, 3917, 2973
*R*_int_	0.027
(sin θ/λ)_max_ (Å^−1^)	0.650

Refinement	
*R*[*F* ^2^ > 2σ(*F* ^2^)], *wR*(*F* ^2^), *S*	0.046, 0.159, 1.04
No. of reflections	3917
No. of parameters	235
H-atom treatment	H atoms treated by a mixture of independent and constrained refinement
Δρ_max_, Δρ_min_ (e Å^−3^)	0.27, −0.38

Computer programs: *APEX2* and *SAINT* (Bruker, 2009 ▸), *SHELXTL* (Sheldrick, 2008 ▸) and *SHELXL2014* (Sheldrick, 2015 ▸).

## supplementary crystallographic information

### Crystal data

**Table:**

C_23_H_14_ClFO	*Z* = 2
*M**_r_* = 360.79	*F*(000) = 372
Triclinic, *P*1	*D*_x_ = 1.383 Mg m^−^^3^
*a* = 9.2846 (9) Å	Mo *K*α radiation, λ = 0.71073 Å
*b* = 9.8777 (10) Å	Cell parameters from 4550 reflections
*c* = 10.3624 (11) Å	θ = 2.5–29.4°
α = 94.364 (2)°	µ = 0.24 mm^−^^1^
β = 113.3517 (19)°	*T* = 296 K
γ = 92.866 (2)°	Plate, yellow
*V* = 866.63 (15) Å^3^	0.43 × 0.39 × 0.11 mm

### Data collection

**Table:**

Bruker SMART APEXII DUO CCD diffractometer	2973 reflections with *I* > 2σ(*I*)
Radiation source: fine-focus sealed tube	*R*_int_ = 0.027
φ and ω scans	θ_max_ = 27.5°, θ_min_ = 2.1°
Absorption correction: multi-scan (*SADABS*; Bruker, 2009)	*h* = −12→12
*T*_min_ = 0.905, *T*_max_ = 0.974	*k* = −12→12
15408 measured reflections	*l* = −13→13
3917 independent reflections	

### Refinement

**Table:**

Refinement on *F*^2^	Primary atom site location: structure-invariant direct methods
Least-squares matrix: full	Secondary atom site location: difference Fourier map
*R*[*F*^2^ > 2σ(*F*^2^)] = 0.046	Hydrogen site location: inferred from neighbouring sites
*wR*(*F*^2^) = 0.159	H atoms treated by a mixture of independent and constrained refinement
*S* = 1.04	*w* = 1/[σ^2^(*F*_o_^2^) + (0.0894*P*)^2^ + 0.1829*P*] where *P* = (*F*_o_^2^ + 2*F*_c_^2^)/3
3917 reflections	(Δ/σ)_max_ = 0.001
235 parameters	Δρ_max_ = 0.27 e Å^−^^3^
0 restraints	Δρ_min_ = −0.38 e Å^−^^3^

### Special details

**Table:**

Geometry. All esds (except the esd in the dihedral angle between two l.s. planes) are estimated using the full covariance matrix. The cell esds are taken into account individually in the estimation of esds in distances, angles and torsion angles; correlations between esds in cell parameters are only used when they are defined by crystal symmetry. An approximate (isotropic) treatment of cell esds is used for estimating esds involving l.s. planes.
Refinement. Refinement of F^2^ against ALL reflections. The weighted R-factor wR and goodness of fit S are based on F^2^, conventional R-factors R are based on F, with F set to zero for negative F^2^. The threshold expression of F^2^ > 2sigma(F^2^) is used only for calculating R-factors(gt) etc. and is not relevant to the choice of reflections for refinement. R-factors based on F^2^ are statistically about twice as large as those based on F, and R- factors based on ALL data will be even larger.

### Fractional atomic coordinates and isotropic or equivalent isotropic displacement parameters (Å^2^)

**Table:**

	*x*	*y*	*z*	*U*_iso_*/*U*_eq_	
F1	0.34800 (14)	0.90969 (12)	0.72673 (15)	0.0699 (4)	
Cl1	0.88071 (6)	0.82117 (5)	1.09430 (6)	0.0677 (2)	
O1	0.52128 (16)	0.43191 (13)	0.84772 (16)	0.0550 (4)	
C1	0.4948 (2)	0.95649 (18)	0.8188 (2)	0.0458 (4)	
C2	0.5336 (3)	1.09407 (19)	0.8323 (2)	0.0550 (5)	
H2A	0.4608	1.1517	0.7807	0.066*	
C3	0.6821 (3)	1.14449 (19)	0.9237 (2)	0.0570 (5)	
H3A	0.7116	1.2372	0.9327	0.068*	
C4	0.7881 (2)	1.05996 (19)	1.0024 (2)	0.0526 (5)	
H4A	0.8886	1.0950	1.0648	0.063*	
C5	0.7438 (2)	0.92195 (17)	0.98776 (19)	0.0429 (4)	
C6	0.5955 (2)	0.86314 (16)	0.89285 (17)	0.0380 (4)	
C7	0.5552 (2)	0.71650 (16)	0.87498 (19)	0.0404 (4)	
H7A	0.6308	0.6670	0.9364	0.048*	
C8	0.4263 (2)	0.64341 (17)	0.7838 (2)	0.0434 (4)	
H8A	0.3445	0.6871	0.7217	0.052*	
C9	0.4112 (2)	0.49374 (17)	0.77988 (19)	0.0398 (4)	
C10	0.2559 (2)	0.41801 (16)	0.68598 (18)	0.0382 (4)	
C11	0.2509 (2)	0.31627 (17)	0.58084 (18)	0.0417 (4)	
C12	0.3822 (3)	0.2912 (2)	0.5479 (2)	0.0565 (5)	
H12A	0.4763	0.3456	0.5947	0.068*	
C13	0.3721 (3)	0.1887 (3)	0.4489 (3)	0.0718 (7)	
H13A	0.4600	0.1736	0.4295	0.086*	
C14	0.2325 (4)	0.1053 (3)	0.3755 (3)	0.0800 (8)	
H14A	0.2291	0.0344	0.3096	0.096*	
C15	0.1029 (3)	0.1272 (3)	0.3997 (2)	0.0700 (6)	
H15A	0.0103	0.0718	0.3496	0.084*	
C16	0.1060 (2)	0.23471 (19)	0.50155 (19)	0.0496 (4)	
C17	−0.0258 (2)	0.2597 (2)	0.5264 (2)	0.0551 (5)	
H17A	−0.1191	0.2055	0.4748	0.066*	
C18	−0.0244 (2)	0.3629 (2)	0.6260 (2)	0.0479 (4)	
C19	−0.1599 (2)	0.3878 (3)	0.6524 (3)	0.0683 (6)	
H19A	−0.2546	0.3364	0.5986	0.082*	
C20	−0.1546 (3)	0.4844 (3)	0.7537 (3)	0.0756 (7)	
H20A	−0.2455	0.5004	0.7677	0.091*	
C21	−0.0121 (3)	0.5609 (2)	0.8381 (3)	0.0679 (6)	
H21A	−0.0092	0.6263	0.9087	0.081*	
C22	0.1212 (2)	0.5408 (2)	0.8184 (2)	0.0528 (5)	
H22A	0.2147	0.5916	0.8769	0.063*	
C23	0.1206 (2)	0.44299 (16)	0.70929 (18)	0.0397 (4)	

### Atomic displacement parameters (Å^2^)

**Table:**

	*U*^11^	*U*^22^	*U*^33^	*U*^12^	*U*^13^	*U*^23^
F1	0.0491 (7)	0.0501 (7)	0.0787 (9)	0.0030 (5)	−0.0066 (6)	0.0012 (6)
Cl1	0.0519 (3)	0.0535 (3)	0.0713 (4)	−0.0034 (2)	−0.0026 (3)	0.0099 (2)
O1	0.0412 (7)	0.0393 (7)	0.0702 (9)	0.0012 (5)	0.0087 (6)	−0.0007 (6)
C1	0.0465 (10)	0.0396 (9)	0.0443 (9)	0.0005 (7)	0.0124 (8)	−0.0026 (7)
C2	0.0695 (13)	0.0368 (9)	0.0554 (11)	0.0083 (9)	0.0216 (10)	0.0036 (8)
C3	0.0770 (14)	0.0323 (9)	0.0588 (12)	−0.0059 (9)	0.0269 (11)	−0.0033 (8)
C4	0.0562 (11)	0.0411 (9)	0.0514 (11)	−0.0150 (8)	0.0166 (9)	−0.0070 (8)
C5	0.0447 (9)	0.0392 (8)	0.0405 (9)	−0.0053 (7)	0.0145 (7)	−0.0009 (7)
C6	0.0423 (9)	0.0335 (8)	0.0377 (8)	−0.0024 (6)	0.0170 (7)	−0.0011 (6)
C7	0.0403 (9)	0.0334 (8)	0.0452 (9)	−0.0010 (7)	0.0161 (7)	0.0006 (7)
C8	0.0388 (9)	0.0353 (8)	0.0504 (10)	−0.0022 (7)	0.0128 (8)	0.0026 (7)
C9	0.0368 (8)	0.0360 (8)	0.0455 (9)	−0.0032 (7)	0.0173 (7)	−0.0026 (7)
C10	0.0376 (8)	0.0336 (8)	0.0403 (8)	−0.0034 (6)	0.0137 (7)	0.0011 (6)
C11	0.0439 (9)	0.0408 (9)	0.0368 (8)	−0.0021 (7)	0.0136 (7)	0.0007 (7)
C12	0.0575 (12)	0.0626 (12)	0.0523 (11)	−0.0026 (10)	0.0281 (10)	−0.0063 (9)
C13	0.0805 (17)	0.0812 (16)	0.0618 (14)	0.0067 (13)	0.0405 (13)	−0.0115 (12)
C14	0.101 (2)	0.0781 (16)	0.0578 (14)	−0.0017 (15)	0.0358 (14)	−0.0256 (12)
C15	0.0773 (15)	0.0670 (14)	0.0508 (12)	−0.0131 (12)	0.0171 (11)	−0.0213 (10)
C16	0.0523 (11)	0.0490 (10)	0.0376 (9)	−0.0053 (8)	0.0102 (8)	−0.0034 (7)
C17	0.0403 (10)	0.0614 (12)	0.0475 (10)	−0.0108 (8)	0.0043 (8)	−0.0053 (9)
C18	0.0357 (9)	0.0537 (10)	0.0470 (10)	−0.0008 (7)	0.0096 (8)	0.0051 (8)
C19	0.0352 (10)	0.0876 (16)	0.0737 (15)	−0.0047 (10)	0.0160 (10)	−0.0008 (13)
C20	0.0488 (12)	0.0917 (18)	0.0939 (19)	0.0084 (12)	0.0379 (13)	0.0001 (15)
C21	0.0670 (14)	0.0652 (13)	0.0828 (16)	0.0050 (11)	0.0444 (13)	−0.0055 (12)
C22	0.0486 (10)	0.0475 (10)	0.0636 (12)	−0.0039 (8)	0.0273 (9)	−0.0080 (9)
C23	0.0383 (8)	0.0362 (8)	0.0422 (9)	−0.0005 (7)	0.0140 (7)	0.0040 (7)

### Geometric parameters (Å, º)

**Table:**

F1—C1	1.350 (2)	C12—C13	1.358 (3)
Cl1—C5	1.734 (2)	C12—H12A	0.9300
O1—C9	1.212 (2)	C13—C14	1.398 (4)
C1—C2	1.371 (3)	C13—H13A	0.9300
C1—C6	1.392 (3)	C14—C15	1.347 (4)
C2—C3	1.367 (3)	C14—H14A	0.9300
C2—H2A	0.9300	C15—C16	1.430 (3)
C3—C4	1.371 (3)	C15—H15A	0.9300
C3—H3A	0.9300	C16—C17	1.377 (3)
C4—C5	1.383 (2)	C17—C18	1.391 (3)
C4—H4A	0.9300	C17—H17A	0.9300
C5—C6	1.400 (2)	C18—C19	1.419 (3)
C6—C7	1.457 (2)	C18—C23	1.432 (2)
C7—C8	1.326 (2)	C19—C20	1.348 (4)
C7—H7A	0.9300	C19—H19A	0.9300
C8—C9	1.474 (2)	C20—C21	1.403 (4)
C8—H8A	0.9300	C20—H20A	0.9300
C9—C10	1.501 (2)	C21—C22	1.353 (3)
C10—C23	1.401 (2)	C21—H21A	0.9300
C10—C11	1.410 (2)	C22—C23	1.429 (3)
C11—C12	1.418 (3)	C22—H22A	0.9300
C11—C16	1.431 (2)		
			
F1—C1—C2	116.89 (17)	C11—C12—H12A	119.6
F1—C1—C6	118.40 (15)	C12—C13—C14	121.4 (2)
C2—C1—C6	124.72 (18)	C12—C13—H13A	119.3
C3—C2—C1	118.30 (19)	C14—C13—H13A	119.3
C3—C2—H2A	120.9	C15—C14—C13	120.3 (2)
C1—C2—H2A	120.9	C15—C14—H14A	119.9
C2—C3—C4	120.80 (17)	C13—C14—H14A	119.9
C2—C3—H3A	119.6	C14—C15—C16	120.8 (2)
C4—C3—H3A	119.6	C14—C15—H15A	119.6
C3—C4—C5	119.26 (18)	C16—C15—H15A	119.6
C3—C4—H4A	120.4	C17—C16—C15	121.58 (19)
C5—C4—H4A	120.4	C17—C16—C11	119.60 (17)
C4—C5—C6	122.82 (17)	C15—C16—C11	118.81 (19)
C4—C5—Cl1	117.09 (14)	C16—C17—C18	122.30 (17)
C6—C5—Cl1	120.08 (13)	C16—C17—H17A	118.8
C1—C6—C5	114.05 (15)	C18—C17—H17A	118.8
C1—C6—C7	124.47 (16)	C17—C18—C19	122.26 (18)
C5—C6—C7	121.48 (16)	C17—C18—C23	118.89 (17)
C8—C7—C6	129.39 (17)	C19—C18—C23	118.79 (18)
C8—C7—H7A	115.3	C20—C19—C18	121.4 (2)
C6—C7—H7A	115.3	C20—C19—H19A	119.3
C7—C8—C9	120.76 (17)	C18—C19—H19A	119.3
C7—C8—H8A	119.6	C19—C20—C21	120.1 (2)
C9—C8—H8A	119.6	C19—C20—H20A	120.0
O1—C9—C8	121.54 (15)	C21—C20—H20A	120.0
O1—C9—C10	120.20 (15)	C22—C21—C20	121.0 (2)
C8—C9—C10	118.24 (15)	C22—C21—H21A	119.5
C23—C10—C11	120.91 (15)	C20—C21—H21A	119.5
C23—C10—C9	119.90 (14)	C21—C22—C23	121.0 (2)
C11—C10—C9	119.07 (15)	C21—C22—H22A	119.5
C10—C11—C12	123.31 (16)	C23—C22—H22A	119.5
C10—C11—C16	118.82 (16)	C10—C23—C22	122.97 (16)
C12—C11—C16	117.86 (16)	C10—C23—C18	119.40 (16)
C13—C12—C11	120.8 (2)	C22—C23—C18	117.55 (16)
C13—C12—H12A	119.6		
			
F1—C1—C2—C3	−179.33 (18)	C11—C12—C13—C14	0.5 (4)
C6—C1—C2—C3	0.7 (3)	C12—C13—C14—C15	1.5 (4)
C1—C2—C3—C4	−1.6 (3)	C13—C14—C15—C16	−0.7 (4)
C2—C3—C4—C5	0.5 (3)	C14—C15—C16—C17	179.2 (2)
C3—C4—C5—C6	1.7 (3)	C14—C15—C16—C11	−1.9 (4)
C3—C4—C5—Cl1	−177.92 (16)	C10—C11—C16—C17	1.9 (3)
F1—C1—C6—C5	−178.72 (16)	C12—C11—C16—C17	−177.37 (19)
C2—C1—C6—C5	1.2 (3)	C10—C11—C16—C15	−177.00 (19)
F1—C1—C6—C7	1.9 (3)	C12—C11—C16—C15	3.7 (3)
C2—C1—C6—C7	−178.11 (18)	C15—C16—C17—C18	179.4 (2)
C4—C5—C6—C1	−2.4 (2)	C11—C16—C17—C18	0.5 (3)
Cl1—C5—C6—C1	177.15 (13)	C16—C17—C18—C19	−179.5 (2)
C4—C5—C6—C7	176.95 (17)	C16—C17—C18—C23	−2.3 (3)
Cl1—C5—C6—C7	−3.5 (2)	C17—C18—C19—C20	177.0 (2)
C1—C6—C7—C8	4.7 (3)	C23—C18—C19—C20	−0.1 (4)
C5—C6—C7—C8	−174.62 (18)	C18—C19—C20—C21	−1.5 (4)
C6—C7—C8—C9	177.73 (16)	C19—C20—C21—C22	1.0 (4)
C7—C8—C9—O1	−8.3 (3)	C20—C21—C22—C23	1.2 (4)
C7—C8—C9—C10	173.29 (16)	C11—C10—C23—C22	177.24 (17)
O1—C9—C10—C23	120.22 (19)	C9—C10—C23—C22	1.3 (3)
C8—C9—C10—C23	−61.4 (2)	C11—C10—C23—C18	0.7 (3)
O1—C9—C10—C11	−55.8 (2)	C9—C10—C23—C18	−175.30 (15)
C8—C9—C10—C11	122.59 (18)	C21—C22—C23—C10	−179.4 (2)
C23—C10—C11—C12	176.76 (17)	C21—C22—C23—C18	−2.8 (3)
C9—C10—C11—C12	−7.2 (3)	C17—C18—C23—C10	1.7 (3)
C23—C10—C11—C16	−2.5 (3)	C19—C18—C23—C10	178.96 (19)
C9—C10—C11—C16	173.54 (16)	C17—C18—C23—C22	−175.05 (19)
C10—C11—C12—C13	177.7 (2)	C19—C18—C23—C22	2.2 (3)
C16—C11—C12—C13	−3.0 (3)		

### Hydrogen-bond geometry (Å, º)

Cg1 is the centroid of the C1–C6 ring.

**Table:**

*D*—H···*A*	*D*—H	H···*A*	*D*···*A*	*D*—H···*A*
C8—H8*A*···F1	0.93	2.19	2.808 (2)	123
C17—H17*A*···F1^i^	0.93	2.46	3.353 (2)	161
C14—H14*A*···*Cg*1^ii^	0.93	2.99	3.712 (3)	136

Symmetry codes: (i) −*x*, −*y*+1, −*z*+1; (ii) −*x*+1, −*y*+1, −*z*+1.
